# Patterns of Male Infertility at the Port Sudan Maternity Hospital

**DOI:** 10.7759/cureus.82290

**Published:** 2025-04-15

**Authors:** Mohammed Ali Saad Ali, Lamia Hassan Sluman Altib, Eithar M Ali, Saja Salah Mohamed Almahdi, Hadeel Abdelseid

**Affiliations:** 1 Obstetrics and Gynecology, Faculty of Medicine, Red Sea University, Port Sudan, SDN; 2 Faculty of Medicine, University of Khartoum, Khartoum, SDN; 3 Obstetrics and Gynecology, University of Khartoum, Khartoum, SDN

**Keywords:** male infertility, oligospermia, risk factors, sexual dysfunction, sperm abnormalities

## Abstract

Background: Male factor infertility is a condition with psychological, economic, social, family, and medical implications.

Objective: To determine the pattern of male infertility at the Port Sudan Maternity Hospital.

Methods: This was an observational descriptive cross-sectional hospital-based study. The study was conducted in the Department of Obstetrics and Gynecology at the Port Sudan Maternity Hospital during the period August 2021 to March 2022 and included all male partners of infertile couples referred for infertility evaluation. Male factor infertility was identified based on standard semen analysis results. The study sample was 114 males who fulfilled the inclusion criteria of the study. Data was collected using a questionnaire that was filled out by patients after taking informed consent from them.

Results: The rate of male infertility was 2.9%. The type of infertility was primary in 60 (52.6%) of the patients and secondary in 54 (47.4%). The patterns of male infertility were abnormal motility and sperm count in 28 (24.6%), abnormal sperm count in 26 (22.8%), abnormal sperm morphology in 20 (17.5%), abnormal sperm motility and morphology in 21 (18.4%), and abnormal sperm motility in 19 (16.7%). The common causes and risk factors of male infertility in this study were sexual problems such as impotence (17, 14.9%), diabetes mellitus (22, 19.3%), positive past history of infections including urinary tract infection (14, 12.3%) and syphilis (2, 1.7%), diabetes medications (20, 17.5%), and exposure to chemicals or physical agents (28, 24.6%). Lifestyle risk factors were smoking (31, 27.2%), alcohol use (10, 8.8%), and substance abuse (2, 1.8%).

Conclusion: The study concluded that the male infertility rate in the study area was within the rate reported by previous regional and international studies. The common patterns of male infertility were abnormal count (oligospermia) and abnormal motility (asthenozoospermia).

## Introduction

Infertility and impaired fecundity have been persistent concerns throughout history and remain significant clinical issues today, affecting approximately 8-12% of couples worldwide. The World Health Organization (WHO) defines infertility as a disease of the reproductive system characterized by the failure to achieve clinical pregnancy after 12 months or more of regular, unprotected sexual intercourse in women under 35 years and after six months in women over 35 years [1]. Estimates suggest that approximately 72.4 million couples globally experience fertility problems, with 60.5 million currently affected by infertility. Male factors account for approximately 40-50% of all infertility cases [2].

Male infertility refers to a man's inability to cause pregnancy in a fertile female. It is typically characterized by alterations in sperm concentration, motility, or morphology below the WHO standards. Diagnosis often involves at least two semen analyses performed one to four weeks apart [3]. Semen analysis remains the most useful diagnostic tool, with a sensitivity of 89.6%, detecting nine out of 10 men with genuine infertility issues [4]. The causes of male infertility can be categorized into pre-testicular, testicular, and post-testicular factors. Pre-testicular causes include hormonal imbalances and systemic conditions. Testicular causes involve genetic defects, infections, and environmental factors. Post-testicular causes are associated with sperm transport disorders, such as vas deferens obstruction [5,6]. Despite extensive diagnostic efforts, approximately 25% of male infertility cases remain idiopathic, where no clear cause is identified [7].

Several lifestyle and environmental factors contribute to male infertility, including smoking, alcohol consumption, obesity, radiation exposure, and nutritional deficiencies. For example, hypogonadism, a condition involving inadequate testosterone production, accounts for 1-2% of cases, while primary testicular failure represents 10-15%. Post-testicular defects and seminiferous tubule dysfunction, including Y chromosome microdeletions, account for 10-20% and 60-80%, respectively [8,9]. Molecular biology techniques have provided insights into genetic causes of male infertility, such as Y chromosome deletions and mutations. The WHO recently redefined oligospermia as a sperm concentration below 15 million sperm/ml, lowering the threshold from the previous 20 million sperm/ml standard [10].

Pre-testicular causes of oligospermia include hormonal imbalances and systemic illnesses such as diabetes and obesity, which can impair sperm production [11]. Testicular factors include age-related decline, genetic abnormalities like Klinefelter syndrome, and exposure to environmental toxins [12,13]. Post-testicular causes, such as obstruction of the vas deferens, can result from genetic conditions like cystic fibrosis or infections [14]. Unexplained (idiopathic) male infertility accounts for approximately 30% of cases, with contributing factors including environmental pollutants, mitochondrial dysfunction, and subtle hormonal changes [15]. Obesity has been linked to a twofold increased risk of oligospermia and azoospermia [16]. Genetic factors, such as BRCA2 mutations, have also been associated with severe oligospermia [17].

Certain medical conditions, including chronic diseases, liver dysfunction, and post-pubertal mumps, may lead to testicular atrophy and impaired sperm production. Treatments for testicular cancer, including chemotherapy and radiation, can cause long-term damage to spermatogenesis [18]. Lifestyle factors play a significant role in male infertility. Cigarette and marijuana use decrease sperm density and motility, while anabolic steroid abuse leads to hypogonadism and structural sperm damage. Heat exposure and spinal cord injuries can also impair sperm production [19,20].

Regional studies highlight the prevalence and contributing factors to male infertility. A study in Khartoum State found that 36.2% of infertility cases were due to male factors, with oligospermia and asthenozoospermia accounting for 16.8% and 17.5%, respectively [21]. Similar patterns were observed in studies from India and Nigeria, where male factor infertility accounted for 25-45% of cases [22,23]. Global data suggest male infertility rates range from 2.5% to 12%, with the highest prevalence in Africa and Central/Eastern Europe [24].

This study aimed to investigate the pattern of male infertility at the Port Sudan Maternity Hospital from August 2021 to March 2022. It sought to determine the frequency of male infertility, identify its common causes, and analyze the types of abnormalities detected in semen analysis.

## Materials and methods

Study setting, population, and sampling

This was an observational, descriptive, cross-sectional hospital-based study conducted in the Department of Obstetrics and Gynecology at Port Sudan Maternity Hospital, a government facility providing 24-hour obstetrics and gynecology services to patients in Port Sudan City and surrounding areas. The study was carried out from August 2021 to March 2022 and included all male partners of infertile couples referred for infertility evaluation. Male factor infertility was identified based on standard semen analysis results, following the WHO criteria, including abnormalities in sperm concentration, motility, or morphology [25]. Men with azoospermia, oligozoospermia, asthenozoospermia, or teratozoospermia were classified accordingly.

The study focused on cases where a male factor was suspected or confirmed, rather than solely unexplained infertility. However, cases with combined or idiopathic (unexplained) infertility were also noted and analyzed separately.

The sample size was calculated using the formula $N = \frac{Z \times P \times Q}{d}$,

where Z = 1.96 (95% confidence level), P = 0.2 (20% estimated prevalence of male infertility), Q = 0.8 (1 - P), and d = 0.05 (margin of error), yielding a required sample size of 114 infertile males. A systematic random sampling technique was used to select participants who met the study criteria. The study variables included age, duration of marriage, occupation, medical history, surgical history, sexual history, fertility history, and medication use.

Data collection

Data collection was conducted using a structured questionnaire through direct interviews (Appendix 1). Data collectors with medical science backgrounds and prior experience in the study area were enrolled after receiving training from the investigator on proper interview techniques and data collection procedures. Data analysis was performed using the IBM SPSS Statistics for Windows, Version 20 (Released 2011; IBM Corp., Armonk, New York, United States), with results presented as frequencies and percentages.

Ethical consideration and approval

Ethical approval was obtained from the Research Ethical Committee at the Red Sea State Ministry of Health, and the Institutional Review Board (IRB) was secured from the Port Sudan Maternity Hospital Research Committee. Participation was voluntary, with respondents providing detailed information about the study's objectives and procedures. Written informed consent was obtained from each participant prior to data collection, and all interviews were conducted in private. Permission to conduct the study was granted by the relevant institutions. The study ensured no harm to participants, prioritized their dignity, and maintained strict confidentiality of all collected data.

## Results

Socio-demographic characteristics and duration and types of infertility

During the study period, a total of 3,864 patients attended the fertility center at Port Sudan Hospital, of whom 114 were infertile males. This corresponds to a male infertility rate of 2.9%. The age distribution of infertile males revealed that the majority (68), with a percentage of 59.6%, were aged between 36 and 50 years, followed by 26 (22.9%) above 50 years and 20 (17.5%) aged between 20 and 35 years. The duration of marriage among the participants was predominantly between five and 10 years, with 59 (51.7%), followed by over 10 years, 31 (27.2%), and less than five years, 24 (21.1%). Regarding the duration of infertility, 49 (43%) of the participants had been infertile for less than five years, 42 (36.8%) between five and 10 years, and 23 (20.2%) for more than 10 years. The most common occupation among infertile males was heavy-duty work, which was 49 (43%), followed by free work, which was 34 (29.8%), and employees were 31 (27.2%). Primary infertility was more common, reported in 60 (52.6%) patients, while secondary infertility was reported in 54 (47.4%) patients (Table 1).

**Table 1:** Demographics and infertility duration

Variable	Frequency (n = 114)	Percentage (%)
Age		
20–35 years	20	17.5
36–50 years	68	59.6
Above 50 years	26	22.9
Duration of Marriage		
Less than five years	24	21.1
Five to 10 years	59	51.7
Above 10 years	31	27.2
Type of Infertility		
Primary	60	52.6
Secondary	54	47.4
Duration of Infertility		
Less than 5 years	49	43.0
5 – 10 years	42	36.8
More than 10 years	23	20.2
Occupation		
Heavy-duty work	49	43.0
Free work	34	29.8
Employee	31	27.2

Past medical, sexual, and treatment history

Of the participants, 66 (57.9%) reported no chronic diseases (Table 2). However, 22 (19.3%) had diabetes mellitus, 17 (14.9%) had hypertension, five (4.4%) had cardiovascular disease (CVD), and four (3.5%) had renal disease. A negative history of infections was reported in 98 (86%) participants. Positive histories included urinary tract infections, which were 14 (12.3%), and syphilis, which was two (1.7%). Most participants (73, 64%) were not on long-term medications. Among those on medications, 20 (17.5%) used diabetes medications, 12 (10.6%) antihypertensives, five (4.4%) CVD medications, and four (3.5%) renal disease medications. Sexual problems, including impotence, were reported by 17 (14.9%) participants. A previous history of infertility treatment was reported in 29 (25.4%) participants.

**Table 2:** Chronic diseases and infections CVD: cardiovascular disease

Variable	Frequency (n = 114)	Percentage (%)
Chronic Diseases		
None	66	57.9
Diabetes mellitus	22	19.3
Hypertension	17	14.9
Cardiovascular disease	5	4.4
Renal disease	4	3.5
Infections		
No history of infections	98	86
Urinary tract infection	14	12.3
Syphilis	2	1.7
Long Term Medication		
No	73	64.0
Diabetes medications	20	17.5
Antihypertensive	12	10.6
CVD medications	5	4.4
Renal disease medications	4	3.5

Surgical and environmental risk factors

Most participants (103, 90.4%) had no previous surgical operations. The reported surgical procedures included hernia repair of five participants (4.4%), fracture repair of three (2.6%), and appendectomy of three participants (2.6%) (Table 3). Environmental exposures to chemicals or physical agents were reported by 28 (24.6%) participants. Lifestyle risk factors included smoking, which was 31 (27.2%), alcohol use was 10 (8.8%), and substance abuse was two (1.8%) (Table 3).

**Table 3:** Risk factors and lifestyle

Variable	Frequency (n = 114)	Percentage (%)
Environmental exposure	28	24.6
Chemicals or physical agents	28	24.6
Lifestyle Risk Factors		
Smoking	31	27.2
Alcohol use	10	8.8
Substance abuse	2	1.8
Previous Surgical Operation		
No	103	90.4
Hernia repair	5	4.4
Fracture repairer	3	2.6
Appendicectomy	3	2.6

Patterns of male infertility 

The patterns of male infertility were distributed as follows: abnormal motility and sperm count, 28 (24.6%); abnormal sperm count, 26 (22.8%); abnormal sperm morphology, 20 (17.5%); abnormal sperm motility and morphology, 21 (18.4%); and abnormal sperm motility, 19 (16.7%) (Table 4).

**Table 4:** Patterns of male infertility

Infertility pattern	Frequency (n = 114)	Percentage (%)
Abnormal motility and count	28	24.6
Abnormal sperm count	26	22.8
Abnormal sperm morphology	20	17.5
Abnormal motility and morphology	21	18.4
Abnormal sperm motility	19	16.7

## Discussion

This study analyzed 114 infertile males at Port Sudan Maternity Hospital between August 2021 and March 2022 to determine the patterns and risk factors associated with male infertility. The overall prevalence of male infertility was 2.9%. Among the participants, 60 (52.6%) had primary infertility, while 54 (47.4%) had secondary infertility. The most common patterns of male infertility included abnormal sperm motility and count (28, 24.6%), isolated abnormal sperm count (26, 22.8%), abnormal sperm morphology (20, 17.5%), combined abnormalities in motility and morphology (21, 18.4%), and isolated abnormal motility (19, 16.7%).

These findings are consistent with global literature on male infertility. Kumar and Singh reported that male factor infertility contributes to approximately 1.8-20% of all infertility cases worldwide, with about 2% of men exhibiting suboptimal sperm parameters. Such abnormalities may involve low sperm concentration (oligozoospermia), poor sperm motility (asthenozoospermia), or abnormal morphology (teratozoospermia) [1,26]. Similar findings were reported by Elussein et al. in Khartoum State, where 62.4% of infertile males presented with primary infertility and 37.6% with secondary infertility. Oligozoospermia and asthenozoospermia were responsible for 16.8% and 17.5% of male infertility cases, respectively. The mean duration of infertility in their study was 5.2 years, indicating a prolonged struggle before patients seek medical care [21]. The findings also align with global patterns reported by Agarwal et al., who highlighted that male infertility rates vary by region, ranging from 2.5% to 12%, with the highest rates observed in Africa and Central/Eastern Europe [24].

The present study identified several medical and lifestyle-related risk factors contributing to male infertility. Sexual dysfunction, particularly impotence, was reported by 17 (14.9%) participants. Diabetes mellitus, a known cause of endocrine-related infertility, was present in 22 (19.3%) cases, while the use of diabetes medications was associated with infertility in 20 (17.5%) participants. A positive history of infections was also prevalent, with 14 (12.3%) participants reporting urinary tract infections and two (1.7%) reporting syphilis. Environmental exposure to chemicals or physical agents, which can impair spermatogenesis, was observed in 28 (24.6%) cases. Lifestyle factors such as smoking (31, 27.2%), alcohol consumption (10, 8.8%), and substance abuse (2, 1.8%) were also linked to male infertility. These findings mirror those of Alam et al., who reported that 38.3% of infertile males had low testosterone levels, and a significant proportion had oligospermia and azoospermia. Additionally, they found that 30% of the cases had erectile dysfunction, which aligns with the findings of this study [22].

Nwajiaku et al. in Nigeria found a high prevalence of secondary infertility (59%), with oligospermia being the most common cause. Male factor infertility alone accounted for 25% of the cases, further emphasizing the significant contribution of male reproductive issues to overall infertility rates [23]. These findings highlight the need for comprehensive evaluation and management strategies that address both male and female factors in infertility cases.

The study by Punab et al. in Ghana categorized male infertility into absolute, severe, and plausible causes. Absolute causes included genetic abnormalities, secondary hypogonadism, and seminal tract obstruction, while severe causes involved oncological diseases and severe sexual dysfunction. Plausible contributing factors included congenital anomalies of the urogenital tract and acquired testicular damage. Their findings suggested that while absolute and severe causes are relatively well understood, a substantial portion of oligozoospermia cases remain idiopathic. This highlights the multifactorial nature of male infertility, where both medical and environmental factors interact to impair reproductive function [27].

The impact of modifiable lifestyle factors on male infertility is well-documented. Durairajanayagam's review emphasized that smoking, obesity, and chronic diseases significantly affect sperm quality. Importantly, many of these factors can be addressed through behavioral modification and better lifestyle choices. The high prevalence of smoking and chemical exposure in our study supports this observation, reinforcing the need for public health interventions targeting these risk factors [28].

This study has several limitations that should be acknowledged. First, the retrospective design restricted us to the information available in patient records, which may have led to incomplete data capture or missing details on relevant risk factors. Additionally, while efforts were made to focus on cases with male infertility, female factors may not have been fully excluded due to limitations in documentation, particularly in the absence of comprehensive couple-based assessments. The diagnosis of isolated male factor infertility may therefore lack full clarity in some cases. Furthermore, as this study was conducted at a single tertiary hospital, the findings may not be generalizable to other settings or populations. Future prospective studies with more comprehensive data collection and broader participant inclusion across multiple centers are recommended to build on these findings and address these limitations.

Recommendations

Addressing male infertility requires a comprehensive approach focusing on both clinical evaluation and public health initiatives. Greater awareness and education about the causes and risk factors of male infertility are essential for early identification and intervention. Public health campaigns should emphasize the importance of reducing modifiable risk factors, such as smoking cessation, limiting alcohol intake, and avoiding exposure to harmful environmental agents. Routine screening for diabetes mellitus and other chronic conditions should be integrated into infertility assessments, particularly for men presenting with sexual dysfunction or a history of infections.

Clinicians should adopt a multidisciplinary approach when evaluating male infertility, including hormonal assessments, semen analysis, and thorough medical histories to identify underlying causes. Future research should focus on elucidating the mechanisms behind unexplained cases of oligozoospermia and exploring targeted therapeutic interventions. Collaboration between healthcare providers, policymakers, and community health organizations is crucial to reducing the burden of male infertility and improving reproductive health outcomes in Sudan.

## Conclusions

The study concluded that the prevalence of male infertility at the Port Sudan Maternity Hospital falls within the range reported by previous regional and international studies. The most common patterns of male infertility were oligospermia and asthenozoospermia, which align with findings from similar studies in other regions. Various medical conditions, including diabetes mellitus and sexual dysfunction, along with modifiable lifestyle factors such as smoking and exposure to environmental toxins, were identified as significant contributors to male infertility. These findings emphasize the need for early detection, targeted interventions, and increased public awareness to mitigate the burden of male infertility.

## Appendices

Appendix 1

Male Infertility Questionnaire

∙ Patient ID: 

∙ Age: 20-35 years, 36-50 years, Above 50 years 

∙ Duration of marriage: Less than five years - Five to 10 years - Above 10 years

∙ Job/Occupation: 

∙ Duration of infertility: ……………….years 

∙ Type of infertility: Primary - Secondary  

∙ Previous infertility treatment: Yes No 

∙ Past medical history of chronic illness 

- No Yes (What is the type, if yes? )

- Diabetes mellitus Yes No 

- Hypertension Yes No 

- Cardiovascular disease Yes No 

- Renal disease Yes No 

- Past history of infection (e.g. mumps) Yes No  

- Urinary tract infection Yes No 

- Syphilis Yes No 

- Long-term medication Yes No  

- Previous surgical operation (e.g., hernia repair) Yes No  

∙ Surgical and environmental risk factors 

- Environmental exposure Yes No 

- Heavy exposure to chemicals or physical agents Yes No 

- Lifestyle risk factors Yes No 

- Smoking Yes No 

- Alcohol intake Yes No 

- Substance abuse Yes No 

- Do you have any sexual problems (e.g. impotence) Yes No 

∙ Semen parameters: 

- Abnormal sperm count alone 

- Abnormal sperm motility alone 

- Abnormal morphology alone 

- Abnormal sperm motility and count 

- Abnormal sperm motility and morphology

## References

[1] Kumar N, Singh AK (2015). Trends of male factor infertility, an important cause of infertility: a review of literature. J Hum Reprod Sci.

[2] Keck C, Gerber-Schafer C, Wilhelm C, Vogelgesang D, Breckwoldt M (1997). Intrauterine insemination for treatment of male infertility. Int J Androl.

[3] Practice Committee of the American Society for Reproductive Medicine (2020). Definitions of infertility and recurrent pregnancy loss: a committee opinion. Fertil Steril.

[4] World Health Organization (1999). WHO. World Health Organization Laboratory Manual for the Examination of Human Semen and Sperm-Cervical Mucus Interaction. The World Health Organization, 1999, WHO, Geneva. World Health Organization Laboratory Manual for the Examination of Human Semen and Sperm-Cervical Mucus Interaction, 4th Ed.

[5] Butt F, Akram Akram (2013). Semen analysis parameters: experience and insight into a male infertility care hospital in Punjab. J Pak Med Assoc.

[6] (2018). WHO: Infertility. https://www.who.int/news-room/fact-sheets/detail/infertility#:~:text=Infertility%20is%20a%20disease%20of,causes%20of%20infertility%20are%20preventable..

[7] Brugh VM, Lipshultz LI (2014). Male factor infertility: evaluation and management. Med Clin North Am.

[8] Hull MG, Glazener CM, Kelly NJ (1985). Population study of causes, treatment, and outcome of infertility. Br Med J (Clin Res Ed).

[9] de Kretser DM (1997). Male infertility. Lancet.

[10] Cooper TG, Noonan E, von Eckardstein S (2010). World Health Organization reference values for human semen characteristics. Hum Reprod Update.

[11] Radwan M, Jurewicz J, Merecz-Kot D (2016). Sperm DNA damage-the effect of stress and everyday life factors. Int J Impot Res.

[12] Leibovitch I, Mor Y (2005). The vicious cycling: bicycling related urogenital disorders. Eur Urol.

[13] Menzies FM, Shepherd MC, Nibbs RJ, Nelson SM (2011). The role of mast cells and their mediators in reproduction, pregnancy and labour. Hum Reprod Update.

[14] Zhang J, Qiu SD, Li SB (2007). Novel mutations in ubiquitin-specific protease 26 gene might cause spermatogenesis impairment and male infertility. Asian J Androl.

[15] Cavallini G (2006). Male idiopathic oligoasthenoteratozoospermia. Asian J Androl.

[16] Sermondade N, Faure C, Fezeu L (2013). BMI in relation to sperm count: an updated systematic review and collaborative meta-analysis. Hum Reprod Update.

[17] Zhoucun A, Zhang S, Yang Y, Ma Y, Zhang W, Lin L (2006). The common variant N372H in BRCA2 gene may be associated with idiopathic male infertility with azoospermia or severe oligozoospermia. Eur J Obstet Gynecol Reprod Biol.

[18] Talebi AR, Khalili MA, Vahidi S, Ghasemzadeh J, Tabibnejad N (2013). Sperm chromatin condensation, DNA integrity, and apoptosis in men with spinal cord injury. J Spinal Cord Med.

[19] El Osta R, Almont T, Diligent C, Hubert N, Eschwège P, Hubert J (2016). Anabolic steroids abuse and male infertility. Basic Clin Androl.

[20] Gundersen TD, Jørgensen N, Andersson AM (2015). Association between use of marijuana and male reproductive hormones and semen quality: a study among 1,215 healthy young men. Am J Epidemiol.

[21] Elussein EA, Magid YM, Omer MM, Adam I (2008). Clinical patterns and major causes of infertility among Sudanese couples. Trop Doct.

[22] Alam J, Choudhary P, Aslam M (2024). Prospective study to evaluate the risk factors associated with male infertility at tertiary care centre. Int Surg J.

[23] Nwajiaku L. A, Mbachu I. I. Ikeako L (2013). Prevalence, clinical pattern and major causes of male infertility in Nnewi, South East Nigeria: a five yearreview. Afrimedic J.

[24] Agarwal A, Mulgund A, Hamada A, Chyatte MR (2015). A unique view on male infertility around the globe. Reprod Biol Endocrinol.

[25] Brannigan RE, Hermanson L, Kaczmarek J, Kim SK, Kirkby E, Tanrikut C (2024). Updates to male infertility: AUA/ASRM guideline (2024). J Urol.

[26] Babakhanzadeh E, Nazari M, Ghasemifar S, Khodadadian A (2020). Some of the factors involved in male infertility: a prospective review. Int J Gen Med.

[27] Punab M, Poolamets O, Paju P (2017). Causes of male infertility: a 9-year prospective monocentre study on 1737 patients with reduced total sperm counts. Hum Reprod.

[28] Durairajanayagam D (2018). Lifestyle causes of male infertility. Arab J Urol.

